# DATA SCIENCE TECHNIQUES TO GAIN NOVEL INSIGHTS ON QUALITY OF CARE: A SCOPING REVIEW OF LONG-TERM CARE FOR OLDER ADULTS

**DOI:** 10.1093/geroni/igad104.2911

**Published:** 2023-12-21

**Authors:** Sil Aarts, Ard Hendriks, Coen Hacking, Hilde Verbeek

**Affiliations:** Maastricht University, Maastricht, Limburg, Netherlands; Maastricht University, Maastricht, Limburg, Netherlands; Maastricht University, Maastricht, Limburg, Netherlands; Maastricht University, Maastricht, Limburg, Netherlands

## Abstract

The increase in powerful computers and technological devices as well as new forms of data analysis such as machine learning have resulted in the widespread availability of data science in healthcare. However, its role in organisations providing long-term care for older adults (LTC) has yet to be systematically synthesized. This study provides a state-of-the-art scoping overview of (1) data science techniques that are used with data accumulated in LTC and for what specific purposes, and (2) the results of employing these techniques in researching the study objective. PubMed and CINHAL was searched using keywords on data science techniques and LTC. The screening-and selection process was carried out by two authors and not limited by research design or publication date. A narrative synthesis was conducted based on the two aims. The search strategy yielded 1,277 studies; 18 were included (the majority conducted in the US and in nursing homes). Machine learning models/algorithms were the most used techniques and were primarily used for researching specific adverse outcomes including the identification of risk factors for falls and the prediction of frailty. This review reveals the limited use of data science techniques on data from LTC facilities. The low number of articles indicates the need for strategies aimed at the effective utilization of data science techniques and evidence of their practical benefits. There is a need for a wider adoption of these techniques in order to exploit data to their full potential and improve the quality of care in LTC by making data-informed decisions.

